# Supplementary material for: OsPIP2;1 Positively Regulates Rice Tolerance to Water Stress Under Coupling of Partial Root-Zone Drying and Nitrogen Forms
Source: Int J Mol Sci. 2025 Oct 8;26(19):9782. doi: 10.3390/ijms26199782 (PMC12525400; doi:10.3390/ijms26199782)

Supplementary materials

For *International Journal of Molecular Sciences*

OsPIP2;1 Positively Regulates Rice Tolerance to Water Stress Under Coupling of Partial Root−Zone Drying and Nitrogen Forms

Chunyi Kuang, Ziying Han, Xiang Zhang, Xiaoyuan Chen*, Zhihong Gao, Yongyong Zhu

Guangdong Provincial Key Laboratory of Utilization and Conservation of Food and Medicinal Resources in the Northern Region, Guangdong Engineering Technology Research Center for Efficient Utilization of Water and Soil Resources in the Northern Region, College of Biology and Agriculture, Shaoguan University, Shaoguan 512005, China

* Correspondence: Xiaoyuan Chen

chenxy88888@sgu.edu.cn; Tel.: +86-15819242815

Materials and Methods

(1) Solution Formulations

1× TAE Buffer: Measure 20 mL of 50× TAE Buffer (Sangon Biotech) and dilute to a final volume of 1 L with pure water.

1% Agarose Gel: Weigh 0.25 g of agarose (Tsingke Biotech) and add it to 25 mL of 1× TAE Buffer. Heat the mixture in a microwave until the solution becomes completely transparent.

N6max Stock Solution (10×): Weigh 56.60 g of KNO_3, 8.00 g of KH_2PO_4, 9.26 g of $(NH_4)_2SO_4$, 3.70 g of $MgSO_4·7H_2O$, and 3.32 g of $CaCl_2·2H_2O$. Dissolve the solids and dilute to a final volume of 2 L with pure water.

N6min Stock Solution (100×): Weigh 0.88 g of $MnSO_4·4H_2O$, 0.30 g of $ZnSO_4·7H_2O$, 0.32 g of H_3BO_3, and 0.16 g of KI. Dissolve the solids and dilute to a final volume of 2 L with pure water.

Fe^{2+}-EDTA Stock Solution (100×): Measure 300 mL of pure water, dissolve 2.78 g of $FeSO_4·7H_2O$ in it, and label this as Solution A. Separately, take 300 mL of pure water, add 3.73 g of Na_2-EDTA·$2H_2O$, and heat the mixture to 70°C until the solid in the beaker is completely dissolved—label this as Solution B. Mix Solution A and Solution B, incubate the combined solution at 70°C for 1 hour, allow it to cool to room temperature, then dilute to a final volume of 1 L with pure water.

Vitamin Stock Solution (100×): Weigh 0.1 g of nicotinic acid, 0.1 g of pyridoxine HCl (VB6), 0.1 g of thiamine HCl (VB1), 0.2 g of glycine, and 10 g of inositol. Dissolve the components and dilute to a final volume of 1 L with pure water.

2,4-D Stock Solution: Weigh 0.1 g of 2,4-dichlorophenoxyacetic acid (2,4-D), add it to 1.0 mL of 1 N KOH, and dilute to a final volume of 100 mL with pure water.

MSmax Stock Solution (10×): Weigh 33.00 g of NH_4NO_3, 3.40 g of KH_2PO_4, 38.00 g of KNO_3, 7.40 g of $MgSO_4·7H_2O$, and 8.80 g of $CaCl_2·2H_2O$. Dissolve the solids and dilute to a final volume of 2 L with pure water.

MSmin Stock Solution (100×): Weigh 4.46 g of $MnSO_4·4H_2O$, 1.72 g of $ZnSO_4·7H_2O$, 1.24 g of H_3BO_3, 0.17 g of KI, 0.05 g of $Na_2MoO_4·2H_2O$, 0.01 g of $CoCl_2·6H_2O$, and

0.01 g of $CuSO_4 \cdot 5H_2O$. Dissolve the solids and dilute to a final volume of 2 L with pure water.

6-BA Stock Solution: Weigh 0.1 g of 6-benzyladenine (6-BA), add it to 1 mL of 1 N KOH, and dilute to a final volume of 100 mL with pure water.

KT Stock Solution: Weigh 0.1 g of kinetin (KT), add it to 1 mL of 1 N KOH, and dilute to a final volume of 100 mL with pure water.

IAA Stock Solution: Weigh 0.1 g of indole-3-acetic acid (IAA), add it to 1 mL of 1 N KOH, and dilute to a final volume of 100 mL with pure water.

NAA Stock Solution: Weigh 0.1 g of 1-naphthaleneacetic acid (NAA), add it to 1 mL of 1 N KOH, and dilute to a final volume of 100 mL with pure water.

200 mM AS Stock Solution: Weigh 0.39 g of acetosyringone (AS), dissolve it in 10 mL of dimethyl sulfoxide (DMSO), and store at -20°C.

250 mg/mL Cn Solution: Weigh 2.5 g of carbenicillin (Cn) [note: the original text has a duplicate "2.5 g" which is corrected here], dissolve it in double-distilled water (ddH_2O), and dilute to a final volume of 10 mL. Store at -20°C.

(2) Medium Formulations

YEB Liquid Medium: Weigh 4 g of yeast extract, 10 g of mannitol, 0.1 g of sodium chloride (NaCl), 0.2 g of $MgSO_4 \cdot 7H_2O$, and 0.5 g of dipotassium hydrogen phosphate (K_2HPO_4). Dissolve the components and dilute to a final volume of 1 L with pure water.

Induction Medium: Add 200 mL of N6max Stock Solution (10×), 20 mL of N6min Stock Solution (100×), 20 mL of Fe^{2+}-EDTA Stock Solution (100×), 20 mL of Vitamin Stock Solution (100×), 5 mL of 2,4-D Stock Solution, 1.2 g of proline (Proline), 1.6 g of casein hydrolysate (CH), 60 g of sucrose (Sucrose), and 6 g of Phytagel. Dilute the mixture to a final volume of 2 L with pure water.

Suspension Medium: Add 25 mL of N6max Stock Solution (10×), 2.50 mL of N6min Stock Solution (100×), 2.50 mL of Fe^{2+}-EDTA Stock Solution (100×), 5 mL of Vitamin Stock Solution (100×), 0.30 g of proline, 0.40 g of casein hydrolysate (CH), 1.25 mL of 2,4-D Stock Solution, and 10 g of sucrose. Dilute to a final volume of 2 L with pure water. Before use, add 10 mL of 50% glucose solution and 500 μL of AS Stock Solution.

Co-Cultivation Medium: Add 25 mL of N6max Stock Solution (10×), 2.50 mL of

N6min Stock Solution (100×), 2.50 mL of Fe^{2+}-EDTA Stock Solution (100×), 5 mL of Vitamin Stock Solution (100×), 1.25 mL of 2,4-D Stock Solution, 0.30 g of proline, 0.40 g of casein hydrolysate (CH), 15 g of sucrose, and 4 g of agar powder. Dilute to a final volume of 2 L with pure water. Before use, add 10 mL of 50% glucose solution and 500 μL of AS Stock Solution.

Selection Medium: Add 50 mL of N6max Stock Solution (10×), 5 mL of N6min Stock Solution (100×), 5 mL of Fe^{2+}-EDTA Stock Solution (100×), 5 mL of Vitamin Stock Solution (100×), 1.25 mL of 2,4-D Stock Solution, 0.30 g of proline, 0.40 g of casein hydrolysate (CH), 15 g of sucrose, and 4 g of agar powder. Dilute to a final volume of 2 L with pure water. Before use, add 500 μL of hygromycin B (Hn) and 1000 μL of Cn Solution.

Differentiation Medium: Add 200 mL of MSmax Stock Solution (10×), 20 mL of MSmin Stock Solution (100×), 20 mL of Fe^{2+}-EDTA Stock Solution (100×), 20 mL of Vitamin Stock Solution (100×), 4 mL of KT Stock Solution, 400 μL of NAA Stock Solution, 1.20 g of proline, 1.60 g of casein hydrolysate (CH), 60 g of D-sorbitol, 60 g of sucrose, and 6 g of Phytagel. Dilute to a final volume of 2 L with pure water.

Rooting Medium: Add 100 mL of MSmax Stock Solution (10×), 10 mL of MSmin Stock Solution (100×), 10 mL of Fe^{2+}-EDTA Stock Solution (100×), 10 mL of Vitamin Stock Solution (100×), 40 g of sucrose, and 6 g of Phytagel. Dilute to a final volume of 2 L with pure water.

Notes on Key Abbreviations

TAE: Tris-Acetate-EDTA (a common buffer for nucleic acid electrophoresis)

AS: Acetosyringone (a reagent that enhances Agrobacterium-mediated transformation)

DMSO: Dimethyl sulfoxide (a solvent for water-insoluble compounds)

ddH_2O: Double-distilled water (high-purity water for laboratory use)

CH: Casein hydrolysate (a nutrient additive for cell culture)

Phytagel: A gelling agent used in plant tissue culture (alternative to agar)

Hn: Hygromycin B (a common selection antibiotic in plant transformation, inferred from context)

Cn: Carbenicillin (an antibiotic used to eliminate Agrobacterium after co-cultivation,

inferred from context)

Table S1. Nutrient solution formulation table of rice seedling.

Elemental	Salts used in nutrient solutions	Elemental concentration （mg/L）	Amount of salts in the nutrient solution （mg/L）		
			A100/N0	A50/N50	A0/N100
N	$(NH_4)_2SO_4/Ca(NO_3)_2\cdot 4H_2O$	40	188.95/0	94.48/168.81	0/337.69
P	$NaH_2PO_4\cdot 2H_2O$	10	50.4	50.4	50.4
K	K_2SO_4	40	89.3	89.3	89.3
Ca	$CaCl_2\cdot 2H_2O/Ca(NO_3)_2\cdot 4H_2O$	57.3	210.20/0	105.11/168.81	0/337.69
Mg	$MgSO_4\cdot 7H_2O$	40	405	405	405
Mn	$MnCl_2\cdot 4H_2O$	0.5	1500	1500	1500
Mo	$Na_2MoO_4\cdot 2H_2O$	0.05	126.25	126.25	126.25
B	H_3BO_3	0.2	934	934	934
Zn	$ZnSO_4\cdot 7H_2O$	0.01	35	35	35
Cu	$CuSO_4\cdot 5H_2O$	0.01	31	31	31

Note: A100/N0, A50/N50 and A0/N100 denote the ratios of NH_4^+ and NO_3^- in the nutrient solution 100/0, 50/50 and 0/100, respectively.

Table S2. Single knockout line target sequencing results of *OsPIP2;1* gene.

Number	Chromosomes	Post-mutation sequence
1	2	CGCTGATCGACGCGGCGGAG(t)CTGGGGTCGTGGTCG(|insert|)||CGCTGATCGACGCGGCGGAG(t)CTGGGGTCGTGGTCG(|insert|)
2	2	GGCGCCGCTGATCGACGCGG------GGGGTCGTGGTCGCT(deletion)||GGCGCCGCTGATCGACGCGGCGGAGTCTGGGGTCG(insertion)
3	2	GGCGCCGCTGATCGACGCGGaGGAtCctGG------GGTCTg(|delete|substitution|)||GGCGCCGCTGATCGACGCGGCGG------GGtGTGG------TCGCGT(|delete|substitution|)
4	2	GGCGCCGCTGATCGACGCGGCGGAG(t)CTGGGGTCGT(|insert|)||GGCGCCGCTGATCGACGCGG------GGGGTCGTGGTCGCTG(|delete|)
5	2	GGCGCCGCTGATCGACGCGGaGGAtCctGG------GGTCTg(|delete|substitution|)||GGCGCCGCTGATCGACGCGGCGG------GGtGTGG------TCGCGT(|delete|substitution|)
6	2	GCGCCGCTGATCGACGCGGCGGAGtgGGG------GgGGTCG(|delete|substitution|)||GCGCCGCTGATCGACGCGGCtcA(g)GCTGGGGTCGTG(|insert|substitution|)
7	2	GGCGCCGCTGATCGACGCGG------aCgGGGGTgGTGGctG(|delete|substitution|)||GGCGCCGCTGATCGACGCGG------tGtGtCGTCgTCGtCG(|delete|substitution|)
8	2	CGCTGATCGACGCGGCGGAG(t)CTGGGGTCGTGGTCG(|insert|)||CGCTGATCGACGCGGCGGAG(t)CTGGGGTCGTGGTCG(|insert|)

Note: The "||" in the sequencing result indicates the two chromosomal mutation sequences separated, and the number of chromosomal mutations is 2, which means that the allele is mutated in both chromosomes.

Figure S1. Identification of *OsPIP2;1* overexpression plant. A1–A11 represent the randomly selected *OsPIP2;1* overexpression plant samples, respectively.

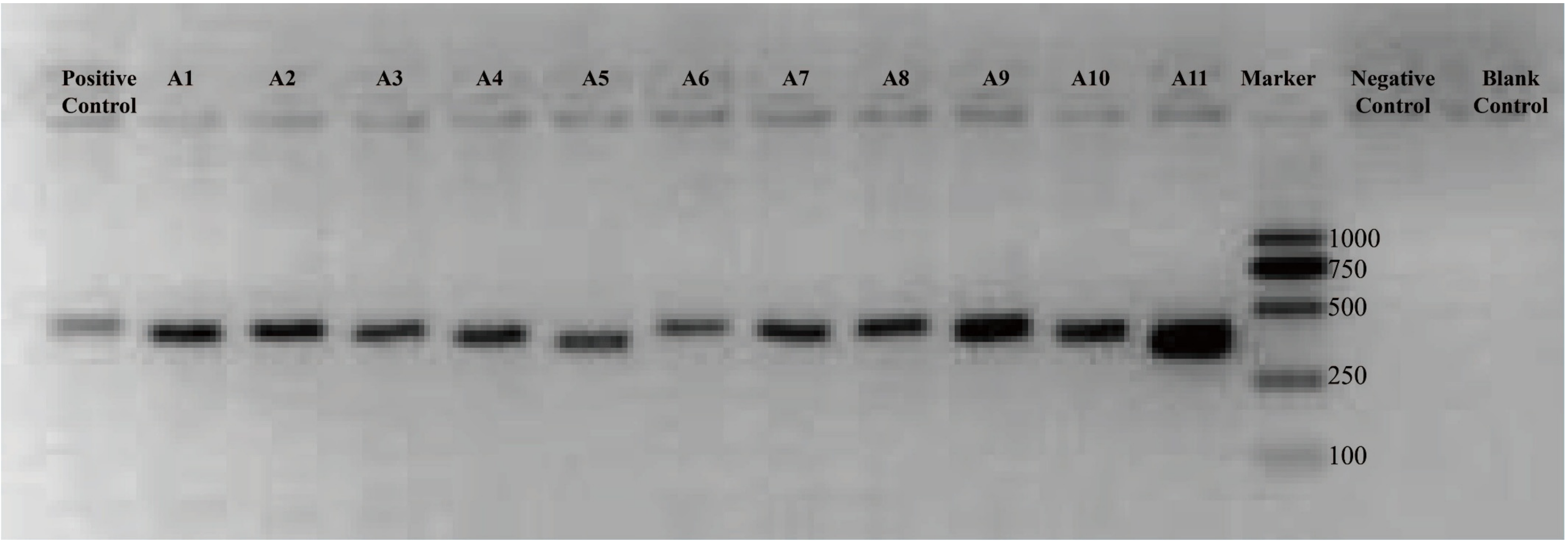

Figure S2. Genetic transformation process of *OsPIP2;1* overexpression and gene knockout plants. Panels A-D show the induction, selection, differentiation, and rooting stages of the overexpression (OE) plants, respectively; Panels E-H show the induction, selection, differentiation, and rooting stages of the gene knockout (KO) plants, respectively.

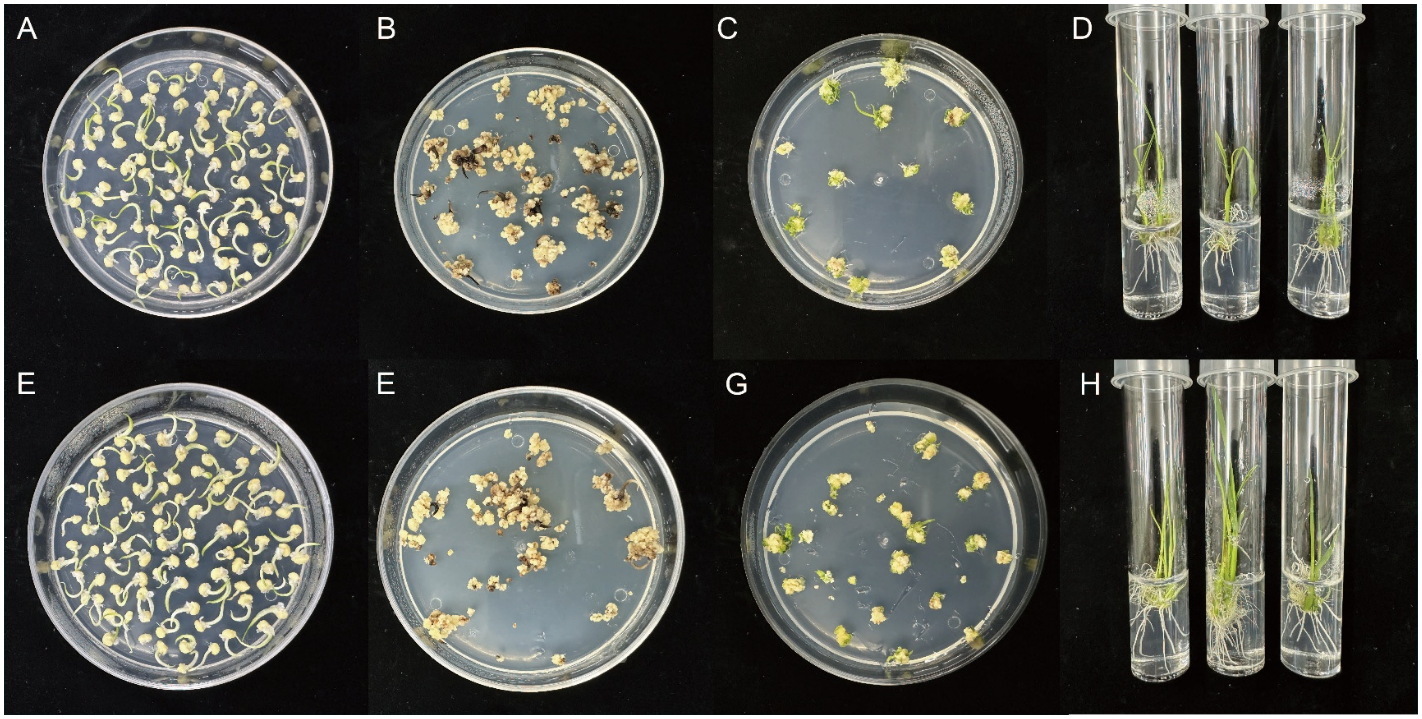

Supplement: Supplementary file 1 [file ijms-26-09782-s001.zip › ijms-3870618-supplementary.pdf]
